# *In Vitro* Activity of the Siderophore Cephalosporin, Cefiderocol, against a Recent Collection of Clinically Relevant Gram-Negative Bacilli from North America and Europe, Including Carbapenem-Nonsusceptible Isolates (SIDERO-WT-2014 Study)

**DOI:** 10.1128/AAC.00093-17

**Published:** 2017-08-24

**Authors:** Meredith A. Hackel, Masakatsu Tsuji, Yoshinori Yamano, Roger Echols, James A. Karlowsky, Daniel F. Sahm

**Affiliations:** a International Health Management Associates, Inc., Schaumburg, Illinois, USA; b Drug Discovery & Disease Research Laboratory, Shionogi & Co., Ltd., Osaka, Japan; c Pharmaceutical Research Division, Shionogi & Co., Ltd., Osaka, Japan; d Clinical Development and Medical Affairs, ID3C, LLC, Easton, Connecticut, USA; e Department of Medical Microbiology, College of Medicine, University of Manitoba, Winnipeg, Manitoba, Canada

**Keywords:** cefiderocol, siderophore, carbapenem-resistant, Chelex 100 resin, Gram-negative bacteria

## Abstract

Cefiderocol (formerly S-649266) is an investigational siderophore cephalosporin. Iron-depleted cation-adjusted Mueller-Hinton broth (ID-CAMHB) was prepared according to the Clinical and Laboratory Standards Institute (CLSI) protocol and used to perform broth microdilution testing of cefiderocol against a 2014-2015 collection of clinical isolates of Gram-negative bacilli from North America (*n* = 4,239) and Europe (*n* = 4,966). The concentrations of cefiderocol inhibiting 90% of isolates tested (MIC_90_s) were 0.5 μg/ml (North America; *n* = 3,007) and 1 μg/ml (Europe; *n* = 3,080) for all isolates of Enterobacteriaceae; 1 μg/ml (North America; *n* = 30) and 4 μg/ml (Europe; *n* = 139) for meropenem-nonsusceptible (MIC ≥ 2 μg/ml) isolates of Enterobacteriaceae; 0.5 μg/ml for both North American (*n* = 765) and European (*n* = 765) isolates of Pseudomonas aeruginosa; 0.5 μg/ml (North America; *n* = 151) and 1 μg/ml (Europe; *n* = 202) for meropenem-nonsusceptible (MIC ≥ 4 μg/ml) isolates of P. aeruginosa; 1 μg/ml for both North American (*n* = 309) and European (*n* = 839) isolates of all Acinetobacter baumannii strains as well as for both North American (*n* = 173) and European (*n* = 595) isolates of meropenem-nonsusceptible A. baumannii; and 0.5μg/ml (North America; *n* = 152) and 0.25 μg/ml (Europe; *n* = 276) for isolates of Stenotrophomonas maltophilia. MICs of cefiderocol were ≤4 μg/ml for 99.9% (6,078/6,087) of all Enterobacteriaceae, 97.0% (164/169) of meropenem-nonsusceptible Enterobacteriaceae, 99.9% (1,529/1,530) of all P. aeruginosa isolates, 100% (353/353) of meropenem-nonsusceptible P. aeruginosa isolates, 97.6% (1,120/1,148) of all A. baumannii isolates, 96.9% (744/768) of meropenem-nonsusceptible A. baumannii isolates, 100% of isolates of S. maltophilia (428/428) and 93.8% of isolates of Burkholderia cepecia (11/12). We conclude that cefiderocol demonstrated potent *in vitro* activity against a recent collection of clinical isolates of commonly encountered Gram-negative bacilli, including carbapenem-nonsusceptible isolates.

## INTRODUCTION

Carbapenems provide effective therapy for patients infected with extended-spectrum β-lactamase (ESBL)-producing Enterobacteriaceae and are considered agents of choice for many Gram-negative infections. Carbapenem-resistant Enterobacteriaceae have emerged or are in the process of emerging in many countries worldwide (1, 2). Carbapenem resistance is also significant among nonfermenting pathogens such as Pseudomonas aeruginosa and Acinetobacter baumannii (3, 4). Observed increases in carbapenem resistance among Enterobacteriaceae and nonfermenters are concerning, as there are few antimicrobial agents available that are safe and effective in the treatment of these infections and the development and distribution of new, more potent agents have not kept pace with increasing and diversifying resistance, particularly for commonly encountered pathogenic Gram-negative bacilli (5, 6). Multidrug-resistant phenotypes, including resistance to cephalosporins, aminoglycosides, and fluoroquinolones, are characteristic of most carbapenem-resistant isolates and present clinicians with difficult decisions to optimize therapy for patients (7). Carbapenem resistance in Gram-negative bacilli may arise by acquisition of class A (e.g., Klebsiella pneumoniae carbapenemase [KPC]), class B (e.g., NDM, IMP, VIM), or class D (e.g., OXA-48) β-lactamases (1, 2, 7–9), by overproduction of chromosomal AmpC, or by acquisition of an ESBL in combination with a porin (e.g., OprD) deficiency or the presence of efflux pumps (9–12).

Cefiderocol, formerly known as S-649226, is a novel siderophore cephalosporin for injection discovered by and currently in clinical development with Shionogi & Co., Ltd., to treat infections caused by carbapenem-resistant Gram-negative bacteria. Cefiderocol has a unique mechanism of cell entry. The cephalosporin moiety of cefiderocol binds primarily to bacterial penicillin binding protein 3 (PBP 3), similar to other cephalosporins, while the catechol moiety at the 3-position side chain of the cephalosporin contributes to forming a chelated complex with ferric iron that facilitates cefiderocol's crossing of the outer membrane of Gram-negative bacilli using the receptor-mediated bacterial iron transport system (13, 14). Bacterial iron transport systems accelerate and increase the influx of cefiderocol to the periplasmic space of Gram-negative bacteria, where its cephalosporin moiety can inhibit cell wall synthesis, thereby enhancing its antimicrobial activity relative to carbapenems, β-lactam/β-lactamase inhibitor combinations, and advanced-generation cephalosporins (14, 15). Cefiderocol has been reported to be active against carbapenem-resistant Gram-negative bacilli harboring various carbapenemases and to be more stable than other β-lactam agents such as ceftazidime, cefepime, and meropenem against class A, B, and D carbapenemases such as KPC, VIM, IMP, NDM, and OXA (15–17). Cefiderocol is also active against ESBL-producing Escherichia coli and Klebsiella pneumoniae (15) as well as against meropenem-resistant P. aeruginosa and A. baumannii (18).

Accurate *in vitro* susceptibility testing of cefiderocol by broth microdilution requires the use of iron-depleted conditions because such conditions induce the production of ferric iron transporters, which are strongly regulated by proximal iron concentrations. Iron-depleted conditions mimic the conditions faced by bacteria infecting human tissues and fluids (15, 17, 19). In January 2016, the Clinical and Laboratory Standards Institute (CLSI) Subcommittee on Antimicrobial Susceptibility Testing approved broth microdilution and disk diffusion methods and quality control MIC ranges for cefiderocol (20). Broth microdilution testing of cefiderocol requires iron-depleted cation-adjusted Mueller-Hinton broth (ID-CAMHB) (20). MICs determined using ID-CAMHB have been shown to be reproducible and to correlate well with *in vivo* efficacy in animal models (21–23). MIC assays for cefiderocol performed in CAMHB with iron concentrations of >0.03 μg/ml show highly variable results and do not correlate with *in vivo* efficacy (22).

The current study, SIDERO-WT-2014, tested a 2014-2015 collection of 9,205 clinical isolates of Gram-negative bacilli from patients in North America and Europe against cefiderocol and comparators using CLSI broth microdilution methodology. This study generated the first *in vitro* surveillance testing data for cefiderocol using the recently approved CLSI broth microdilution MIC determination method (20).

## RESULTS

**FIG 1:**
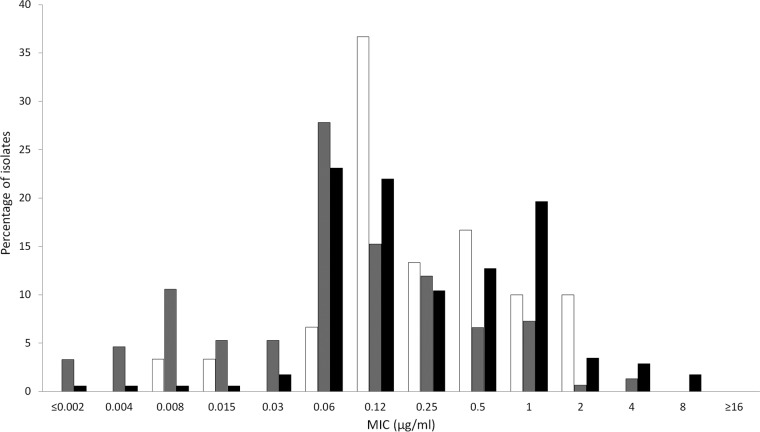
Cefiderocol MIC distributions for meropenem-nonsusceptible Enterobacteriaceae (white bars; *n* = 30), P. aeruginosa (gray bars; *n* = 151), and A. baumannii (black bars; *n* = 173) isolates collected by North American medical center laboratories.

**FIG 2:**
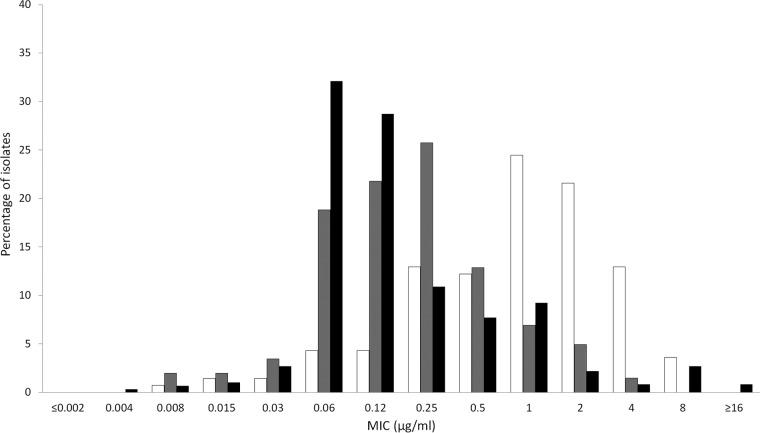
Cefiderocol MIC distributions for meropenem-nonsusceptible Enterobacteriaceae (white bars; *n* = 139), P. aeruginosa (gray bars; *n* = 202), and A. baumannii (black bars; *n* = 595) isolates collected by European medical center laboratories.

**FIG 3:**
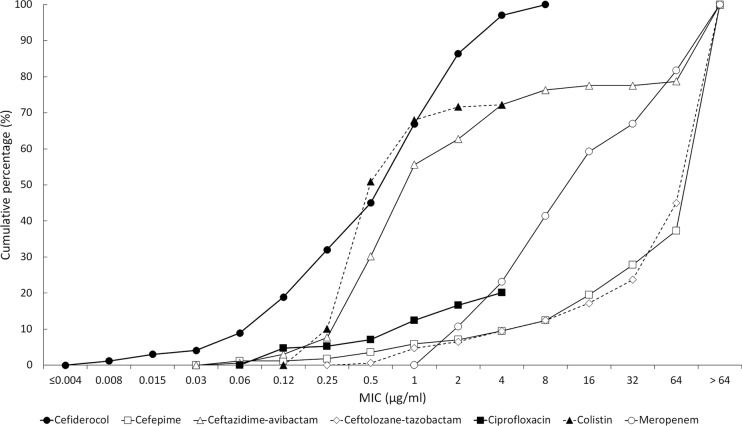
Cumulative MIC susceptibility curves for cefiderocol and comparators against 169 meropenem nonsusceptible Enterobacteriaceae from North American and European medical center laboratories. For colistin and ciprofloxacin, the endpoint of their respective cumulative MIC susceptibility curves represents the highest concentration tested. The remaining tested isolates had MICs higher than their endpoint value.

The *in vitro* activities of cefiderocol and comparators are summarized in Table 1 for the 4,239 isolates collected from North American medical center laboratories and in Table 2 for the 4,966 isolates from European medical center laboratories. The concentrations of antimicrobial agent inhibiting 90% of isolates tested (MIC_90_s) for cefiderocol against Enterobacteriaceae were 0.5 μg/ml (North America, *n* = 3,007 isolates) and 1 μg/ml (Europe, *n* = 3,080 isolates). The MIC range for cefiderocol was ≤0.002 to 8 μg/ml for both sets of isolates, and 99.9% (6,078/6,087) of all Enterobacteriaceae had cefiderocol MICs of ≤4 μg/ml. One isolate of Serratia marcescens from North America had a MIC to cefiderocol of 8 μg/ml, while six isolates of K. pneumoniae, one isolate of Enterobacter aerogenes, and one isolate of S. marcescens from Europe also had a MIC to cefiderocol of 8 μg/ml. Five of the nine isolates with cefiderocol MICs of 8 μg/ml were nonsusceptible to meropenem. Against meropenem-nonsusceptible (MIC ≥ 2 μg/ml) isolates from North America (Fig. 1) and Europe (Fig. 2), MIC_90_ values were 1 μg/ml (*n* = 30) and 4 μg/ml (*n* = 139); 97.0% (164/169) of all meropenem-nonsusceptible Enterobacteriaceae had MICs to cefiderocol of ≤4 μg/ml. In comparison, testing of recently approved antimicrobial agents ceftazidime-avibactam and ceftolozane-tazobactam against the same geographic sets of isolates of meropenem-nonsusceptible Enterobacteriaceae demonstrated MIC_90_ values of 4 and >64 μg/ml, respectively, for isolates from North America (Table 1) and >64 and >64 μg/ml, respectively, for isolates from Europe (Table 2). Against meropenem-susceptible (MIC ≤1 μg/ml) isolates from North America and Europe, MIC_90_ values for cefiderocol were 0.5 μg/ml (*n* = 2,977) and 1 μg/ml (*n* = 2,941); 99.9% (5,914/5,918) of all meropenem-susceptible Enterobacteriaceae had MICs to cefiderocol of ≤4 μg/ml. Figure 3 depicts the cumulative percentages of all meropenem-nonsusceptible isolates of Enterobacteriaceae that were susceptible to increasing concentrations of cefiderocol and its comparators.

**TABLE 1:** *In vitro* activity of cefiderocol and comparators against Gram-negative bacilli (*n* = 4,239) isolated by 50 medical center laboratories in North America from 2014 to 2015

Family/genus/species (no. of isolates)	Antimicrobial agent	MIC (μg/ml)^*a*^			MIC interpretation^*b*^		
		Range	MIC_50_	MIC_90_	% susceptible	% intermediate	% resistant
Enterobacteriaceae (3,007)	Cefiderocol	≤0.002–8	0.06	0.5			
	Cefepime	≤0.06 to >64	≤0.06	0.5	93.7	2.2	4.2
	Ceftazidime-avibactam	≤0.06 to >64	0.12	0.5	99.9	0	0.1
	Ceftolozane-tazobactam	≤0.06 to >64	0.25	1	94.3	1.8	4.0
	Ciprofloxacin	≤0.12 to >8	≤0.12	>8	86.2	1.4	12.3
	Colistin	≤0.25 to >8	0.5	>8	82.4	0	17.6
	Meropenem	≤0.06 to >64	≤0.06	≤0.06	99.0	0.1	0.9
Meropenem-nonsusceptible Enterobacteriaceae (30)	Cefiderocol	0.008–2	0.12	1			
	Cefepime	≤0.06 to >64	16	>64	10.0	23.3	66.7
	Ceftazidime-avibactam	0.12 to >64	1	4	96.7	0	3.3
	Ceftolozane-tazobactam	1 to >64	64	>64	13.3	6.7	80.0
	Ciprofloxacin	≤0.12 to >8	8	>8	20.0	16.7	63.3
	Colistin	≤0.25 to >8	0.5	>8	66.7	0	33.3
	Meropenem	2 to >64	8	64	0	13.3	86.7
Klebsiella spp.^*c*^ (1,010)	Cefiderocol	≤0.002–4	0.06	0.25			
	Cefepime	≤0.06 to >64	≤0.06	0.5	93.9	2.0	4.2
	Ceftazidime-avibactam	≤0.06–8	0.12	0.5	100	0	0
	Ceftolozane-tazobactam	≤0.06 to >64	0.25	1	96.8	0.6	2.6
	Ciprofloxacin	≤0.12 to >8	≤0.12	0.5	92.9	0.8	6.3
	Colistin	≤0.25 to >8	0.5	1	99.4	0	0.6
	Meropenem	≤0.06 to >64	≤0.06	≤0.06	98.4	0.1	1.5
Klebsiella pneumoniae (765)	Cefiderocol	≤0.002–4	0.03	0.5			
	Cefepime	≤0.06 to >64	≤0.06	0.5	92.7	2.2	5.1
	Ceftazidime-avibactam	≤0.06–8	0.12	0.5	100	0	0
	Ceftolozane-tazobactam	≤0.06 to >64	0.25	1	96.3	0.8	2.9
	Ciprofloxacin	≤0.12 to >8	≤0.12	1	91.5	1.1	7.5
	Colistin	≤0.25 to >8	0.5	1	99.4	0	0.7
	Meropenem	≤0.06 to >64	≤0.06	≤0.06	98.0	0.1	1.8
Klebsiella oxytoca (245)	Cefiderocol	≤0.002–2	0.06	0.25			
	Cefepime	≤0.06 to >64	≤0.06	0.12	97.6	1.2	1.2
	Ceftazidime-avibactam	≤0.06–2	0.12	0.25	100	0	0
	Ceftolozane-tazobactam	≤0.06–64	0.25	0.5	98.4	0	1.6
	Ciprofloxacin	≤0.12 to >8	≤0.12	≤0.12	97.1	0	2.9
	Colistin	≤0.25 to >8	0.5	1	99.6	0	0.4
	Meropenem	≤0.06–8	≤0.06	≤0.06	99.6	0	0.4
Escherichia coli (740)	Cefiderocol	≤0.002–2	0.06	0.25			
	Cefepime	≤0.06 to >64	≤0.06	8	88.8	1.8	9.5
	Ceftazidime-avibactam	≤0.06–2	0.12	0.25	100	0	0
	Ceftolozane-tazobactam	≤0.06 to >64	0.25	0.5	97.4	1.0	1.6
	Ciprofloxacin	≤0.12 to >8	≤0.12	>8	66.0	0.1	33.9
	Colistin	≤0.25 to >8	0.5	1	99.7	0	0.3
	Meropenem	≤0.06–4	≤0.06	≤0.06	99.9	0	0.1
Serratia spp.^*d*^ (503)	Cefiderocol	≤0.002–8	0.06	0.25			
	Cefepime	≤0.06 to >64	≤0.06	0.25	98.0	1.2	0.8
	Ceftazidime-avibactam	≤0.06–2	0.12	0.5	100	0	0
	Ceftolozane-tazobactam	0.12 to >64	0.5	1	97.2	1.4	1.4
	Ciprofloxacin	≤0.12 to >8	≤0.12	1	90.3	4.8	5.0
	Colistin	0.5 to >8	>8	>8	5.0	0	95.0
	Meropenem	≤0.06–64	≤0.06	0.12	98.2	0.4	1.4
Serratia marcescens (472)	Cefiderocol	0.004–8	0.06	0.25			
	Cefepime	≤0.06 to >64	≤0.06	0.25	97.9	1.3	0.9
	Ceftazidime-avibactam	≤0.06–2	0.12	0.5	100	0	0
	Ceftolozane-tazobactam	0.12 to >64	0.5	1	97.0	1.5	1.5
	Ciprofloxacin	≤0.12 to >8	≤0.12	2	89.6	5.1	5.3
	Colistin	0.5 to >8	>8	>8	5.1	0	94.9
	Meropenem	≤0.06–64	≤0.06	0.12	98.1	0.4	1.5
Enterobacter spp.^*e*^ (494)	Cefiderocol	0.004–4	0.25	1			
	Cefepime	≤0.06 to >64	≤0.06	1	93.9	4.5	1.6
	Ceftazidime-avibactam	≤0.06–4	0.25	0.5	100	0	0
	Ceftolozane-tazobactam	≤0.06–64	0.25	8	81.8	6.3	11.9
	Ciprofloxacin	≤0.12 to >8	≤0.12	0.25	94.9	1.8	3.2
	Colistin	≤0.25 to >8	0.5	2	91.3	0	8.7
	Meropenem	≤0.06–32	≤0.06	0.12	99.6	0	0.4
Enterobacter aerogenes (238)	Cefiderocol	0.004–4	0.12	0.5			
	Cefepime	≤0.06–16	≤0.06	0.5	97.1	2.5	0.4
	Ceftazidime-avibactam	≤0.06–4	0.25	0.5	100	0	0
	Ceftolozane-tazobactam	≤0.06–16	0.25	4	81.9	10.1	8.0
	Ciprofloxacin	≤0.12 to >8	≤0.12	0.25	97.5	0.4	2.1
	Colistin	≤0.25 to >8	0.5	1	98.3	0	1.7
	Meropenem	≤0.06–8	≤0.06	0.12	99.6	0	0.4
Enterobacter cloacae (213)	Cefiderocol	0.008–4	0.25	1			
	Cefepime	≤0.06 to >64	≤0.06	4	89.2	7.5	3.3
	Ceftazidime-avibactam	≤0.06–4	0.25	1	100	0	0
	Ceftolozane-tazobactam	≤0.06–64	0.5	8	80.3	3.3	16.4
	Ciprofloxacin	≤0.12 to >8	≤0.12	1	92.5	3.3	4.2
	Colistin	≤0.25 to >8	0.5	1	94.8	0	5.2
	Meropenem	≤0.06–32	≤0.06	0.12	99.5	0	0.5
Enterobacter asburiae (30)	Cefiderocol	≤0.06–0.5	0.25	1			
	Cefepime	≤0.06–2	≤0.06	0.25	100	0	0
	Ceftazidime-avibactam	0.12–16	0.25	0.5	100	0	0
	Ceftolozane-tazobactam	≤0.12 to >8	0.25	8	86.7	0	13.3
	Ciprofloxacin	≤0.25 to >8	≤0.12	0.5	93.3	0	6.7
	Colistin	≤0.06–0.25	>8	>8	13.3	0	86.7
	Meropenem	≤0.06–0.5	≤0.06	0.12	100	0	0
Citrobacter spp.^*f*^ (260)	Cefiderocol	≤0.002–2	0.12	0.25			
	Cefepime	≤0.06 to >64	≤0.06	0.25	97.7	1.5	0.8
	Ceftazidime-avibactam	≤0.06 to >64	0.12	0.25	99.2	0	0.8
	Ceftolozane-tazobactam	≤0.06 to >64	0.25	1	93.1	0.8	6.2
	Ciprofloxacin	≤0.12 to >8	≤0.12	0.5	93.9	0.4	5.8
	Colistin	≤0.25–2	0.5	1	100	0	0
	Meropenem	≤0.06–8	≤0.06	≤0.06	99.2	0.4	0.4
Citrobacter freundii (143)	Cefiderocol	≤0.002–1	0.06	0.25			
	Cefepime	≤0.06 to >64	≤0.06	1	96.5	2.1	1.4
	Ceftazidime-avibactam	≤0.06 to >64	0.12	0.5	99.3	0	0.7
	Ceftolozane-tazobactam	≤0.06 to >64	0.25	8	88.1	1.4	10.5
	Ciprofloxacin	≤0.12 to >8	≤0.12	1	91.6	0.7	7.7
	Colistin	≤0.25–2	0.5	1	100	0	0
	Meropenem	≤0.06–8	≤0.06	≤0.06	98.6	0.7	0.7
Citrobacter koseri (99)	Cefiderocol	0.06–2	0.25	0.5			
	Cefepime	≤0.06–0.25	≤0.06	≤0.06	100	0	0
	Ceftazidime-avibactam	≤0.06–0.5	0.12	0.12	100	0	0
	Ceftolozane-tazobactam	≤0.06–1	0.25	0.5	100	0	0
	Ciprofloxacin	≤0.12 to >8	≤0.12	≤0.12	96.0	0	4.0
	Colistin	≤0.25–1	≤0.25	1	100	0	0
	Meropenem	≤0.06–0.12	≤0.06	≤0.06	100	0	0
Pseudomonas aeruginosa (765)	Cefiderocol	≤0.002–8	0.06	0.5			
	Cefepime	≤0.06 to >64	4	16	85.5	8.1	6.4
	Ceftazidime-avibactam	≤0.06 to >64	2	8	98.0	0	2.0
	Ceftolozane-tazobactam	≤0.06 to >64	0.5	2	97.7	1.2	1.2
	Ciprofloxacin	≤0.12 to >8	0.25	8	77.9	7.5	14.6
	Colistin	≤0.25–4	1	2	99.5	0.5	0
	Meropenem	≤0.06 to >64	0.25	8	80.3	6.0	13.7
Meropenem-nonsusceptible Pseudomonas aeruginosa (151)	Cefiderocol	≤0.002–4	0.06	0.5			
	Cefepime	1 to >64	8	32	53.6	21.9	24.5
	Ceftazidime-avibactam	0.5 to >64	4	8	90.7	0	9.3
	Ceftolozane-tazobactam	0.25 to >64	1	4	90.1	4.6	5.3
	Ciprofloxacin	≤0.12 to >8	2	>8	43.7	12.6	43.7
	Colistin	≤0.25–4	1	1	99.3	0.7	0
	Meropenem	4 to >64	8	16	0	30.5	69.5
Acinetobacter baumannii (309)	Cefiderocol	≤0.002–8	0.12	1			
	Cefepime	0.25 to >64	16	64	49.2	20.1	30.7
	Ceftazidime-avibactam	1 to >64	16	>64			
	Ceftolozane-tazobactam	≤0.06 to >64	8	>64			
	Ciprofloxacin	≤0.12 to >8	>8	>8	34.3	0.3	65.4
	Colistin	≤0.25 to >8	0.5	1	94.8	0	5.2
	Meropenem	≤0.06 to >64	8	>64	44.0	1.6	54.4
Meropenem-nonsusceptible Acinetobacter baumannii (173)	Cefiderocol	≤0.002–8	0.25	1			
	Cefepime	4 to >64	32	>64	16.8	31.8	51.5
	Ceftazidime-avibactam	4 to >64	32	>64			
	Ceftolozane-tazobactam	0.5 to >64	16	>64			
	Ciprofloxacin	0.25 to >8	>8	>8	1.7	0	98.3
	Colistin	≤0.25 to >8	0.5	2	91.3	0	8.7
	Meropenem	4 to >64	64	>64	0	2.9	97.1
Stenotrophomonas maltophilia (152)	Cefiderocol	≤0.002–4	0.06	0.5			
	Cefepime	1 to >64	32	>64			
	Ceftazidime-avibactam	0.5 to >64	8	64			
	Ceftolozane-tazobactam	0.12 to >64	8	>64			
	Ciprofloxacin	0.25 to >8	2	>8			
	Colistin	≤0.25 to >8	2	>8			
	Meropenem	1 to >64	>64	>64			
Burkholderia cepacia (6)	Cefiderocol	0.015–16					
	Cefepime	16 to >64					
	Ceftazidime-avibactam	4					
	Ceftolozane-tazobactam	2–16					
	Ciprofloxacin	0.5–8					
	Colistin	>8					
	Meropenem	2–8			83.3	16.7	0

a MIC_50_ and MIC_90_ calculated only for genus or species where >30 isolates were tested. Species of Enterobacteriaceae with <30 isolates were grouped with the overall genus data.

b Blank spaces mean that there are no CLSI, EUCAST, or FDA MIC breakpoints available for this agent.

c The 1,010 isolates of Klebsiella spp. were composed of 765 Klebsiella pneumoniae and 245 Klebsiella oxytoca isolates.

d The 503 isolates of Serratia spp. were composed of 472 Serratia marcescens, 22 Serratia liquefaciens, 4 Serratia ureilytica, 3 Serratia odorifera, 1 Serratia grimesii, and 1 Serratia rubidaea isolates.

e The 494 isolates of Enterobacter spp. were composed of 238 Enterobacter aerogenes, 213 Enterobacter cloacae, 30 Enterobacter asburiae, 10 Enterobacter kobei, 2 Enterobacter ludwigii, and 1 Enterobacter amnigenus isolates.

f The 260 isolates of Citrobacter spp. were composed of 143 Citrobacter freundii, 99 Citrobacter koseri, 10 Citrobacter braakii, 5 Citrobacter amalonaticus, 2 Citrobacter farmeri, and 1 Citrobacter sedlakii isolates.

**TABLE 2:** *In vitro* activity of cefiderocol and comparators against Gram-negative bacilli (*n* = 4,966) isolated by 49 medical center laboratories in Europe from 2014 to 2015

Family/genus/species (no. of isolates)	Antimicrobial agent	MIC (μg/ml)^*a*^			MIC interpretation^*b*^		
		Range	MIC_50_	MIC_90_	% susceptible	% intermediate	% resistant
Enterobacteriaceae (3,080)	Cefiderocol	≤0.002–8	0.12	1			
	Cefepime	≤0.06 to >64	≤0.06	>64	81.6	3.1	15.4
	Ceftazidime-avibactam	≤0.06 to >64	0.12	0.5	98.6	0	1.4
	Ceftolozane-tazobactam	≤0.06 to >64	0.25	8	87.1	2.4	10.5
	Ciprofloxacin	≤0.12 to >8	≤0.12	>8	80.5	1.8	17.8
	Colistin	≤0.25 to >8	0.5	>8	82.0	0	18.0
	Meropenem	≤0.06 to >64	≤0.06	0.12	95.0	0.5	4.1
Meropenem-nonsusceptible Enterobacteriaceae (139)	Cefiderocol	0.008–8	1	4			
	Cefepime	0.25 to >64	>64	>64	6.5	1.4	92.1
	Ceftazidime-avibactam	≤0.06 to >64	1	>64	71.9	0	28.1
	Ceftolozane-tazobactam	0.5 to >64	>64	>64	5.0	2.2	92.8
	Ciprofloxacin	≤0.12 to >8	>8	>8	10.8	1.4	87.8
	Colistin	≤0.25 to >8	1	>8	72.7	0	27.3
	Meropenem	2 to >64	16	>64	0	10.1	89.9
Klebsiella spp.^*c*^ (1,021)	Cefiderocol	≤0.002–8	0.12	2			
	Cefepime	≤0.06 to >64	≤0.06	>64	70.3	3.3	26.4
	Ceftazidime-avibactam	≤0.06 to >64	0.12	1	97.8	0	2.3
	Ceftolozane-tazobactam	≤0.06 to >64	0.25	64	83.0	1.3	15.8
	Ciprofloxacin	≤0.12 to >8	≤0.12	>8	72.3	2.6	25.1
	Colistin	≤0.25 to >8	0.5	1	95.0	0	5.0
	Meropenem	≤0.06 to >64	≤0.06	2	89.9	1.0	9.1
Klebsiella pneumoniae (761)	Cefiderocol	≤0.002–8	0.12	2			
	Cefepime	≤0.06 to >64	0.12	>64	63.1	3.0	33.9
	Ceftazidime-avibactam	≤0.06 to >64	0.25	1	97.1	0	2.9
	Ceftolozane-tazobactam	≤0.06 to >64	0.5	64	78.2	1.3	20.5
	Ciprofloxacin	≤0.12 to >8	≤0.12	>8	64.4	2.8	32.9
	Colistin	≤0.25 to >8	0.5	1	93.3	0	6.7
	Meropenem	≤0.06 to >64	≤0.06	8	86.7	1.3	12.0
Klebsiella oxytoca (260)	Cefiderocol	≤0.002–2	0.03	0.25			
	Cefepime	≤0.06 to >64	≤0.06	1	91.5	4.2	4.2
	Ceftazidime-avibactam	≤0.06 to >64	0.12	0.25	99.6	0	0.4
	Ceftolozane-tazobactam	≤0.06 to >64	0.25	0.5	96.9	1.2	1.9
	Ciprofloxacin	≤0.12 to >8	≤0.12	≤0.12	95.4	2.3	2.3
	Colistin	≤0.25–2	0.5	1	100	0	0
	Meropenem	≤0.06–8	≤0.06	≤0.06	99.2	0	0.8
Escherichia coli (789)	Cefiderocol	≤0.002–4	0.12	0.5			
	Cefepime	≤0.06 to >64	≤0.06	64	81.6	3.6	14.8
	Ceftazidime-avibactam	≤0.06–32	0.12	0.25	99.8	0	0.3
	Ceftolozane-tazobactam	≤0.06 to >64	0.25	0.5	96.5	0.4	3.2
	Ciprofloxacin	≤0.12 to >8	≤0.12	>8	72.9	0.5	26.6
	Colistin	≤0.25 to >8	0.5	1	99.5	0	0.5
	Meropenem	≤0.06–4	≤0.06	≤0.06	99.6	0	0.4
Serratia spp.^*d*^ (493)	Cefiderocol	≤0.002–8	0.12	0.5			
	Cefepime	≤0.06 to >64	0.12	0.5	94.1	1.0	4.9
	Ceftazidime-avibactam	≤0.06 to >64	0.12	0.5	99.2	0	0.8
	Ceftolozane-tazobactam	0.12 to >64	0.5	1	95.3	1.8	2.8
	Ciprofloxacin	≤0.12 to >8	≤0.12	1	93.7	2.2	4.1
	Colistin	0.5 to >8	>8	>8	5.1	0	94.9
	Meropenem	≤0.06–64	≤0.06	0.12	99.0	0	1.0
Serratia marcescens (455)	Cefiderocol	≤0.002–8	0.12	0.5			
	Cefepime	≤0.06 to >64	0.12	0.5	93.6	1.1	5.3
	Ceftazidime-avibactam	≤0.06 to >64	0.12	0.5	99.1	0	0.9
	Ceftolozane-tazobactam	0.25 to >64	0.5	1	95.2	2.0	2.9
	Ciprofloxacin	≤0.12 to >8	≤0.12	1	93.4	2.4	4.2
	Colistin	0.5 to >8	>8	>8	5.5	0	94.5
	Meropenem	≤0.06–64	≤0.06	0.12	98.9	0	1.1
Serratia liquefaciens (33)	Cefiderocol	0.015–0.25	0.06	0.12			
	Cefepime	≤0.06–0.25	≤0.06	0.12	100	0	0
	Ceftazidime-avibactam	0.12–1	0.25	0.5	100	0	0
	Ceftolozane-tazobactam	0.25–1	0.5	1	100	0	0
	Ciprofloxacin	≤0.12–0.5	≤0.12	≤0.12	100	0	0
	Colistin	>8	>8	>8	0	0	100
	Meropenem	≤0.06–0.25	≤0.06	0.12	100	0	0
Enterobacter spp.^*e*^ (530)	Cefiderocol	0.008–8	0.25	1			
	Cefepime	≤0.06 to >64	≤0.06	16	85.7	4.2	10.2
	Ceftazidime-avibactam	≤0.06 to >64	0.25	0.5	97.9	0	2.1
	Ceftolozane-tazobactam	≤0.06–64	0.5	8	75.7	7.6	16.8
	Ciprofloxacin	≤0.12 to >8	≤0.12	1	90.0	2.1	7.9
	Colistin	≤0.25 to >8	0.5	1	94.3	0	5.7
	Meropenem	≤0.06–64	≤0.06	0.12	96.2	0.6	3.2
Enterobacter cloacae (301)	Cefiderocol	0.008–4	0.25	1			
	Cefepime	≤0.06 to >64	0.12	32	78.4	6.6	15.0
	Ceftazidime-avibactam	≤0.06 to >64	0.25	1	96.4	0	3.7
	Ceftolozane-tazobactam	≤0.06 to >64	0.5	16	74.1	3.0	22.9
	Ciprofloxacin	≤0.12 to >8	≤0.12	8	85.1	2.7	12.3
	Colistin	≤0.25 to >8	0.5	1	95.7	0	4.3
	Meropenem	≤0.06–64	≤0.06	0.25	93.7	1.0	5.3
Enterobacter aerogenes (204)	Cefiderocol	0.015–8	0.12	0.5			
	Cefepime	≤0.06 to >64	≤0.06	1	94.6	1.0	4.4
	Ceftazidime-avibactam	≤0.06–8	0.25	0.5	100	0	0
	Ceftolozane-tazobactam	0.12–32	0.5	4	77.0	14.2	8.8
	Ciprofloxacin	≤0.12 to >8	≤0.12	0.25	96.1	1.5	2.5
	Colistin	≤0.25 to >8	0.5	1	98.5	0	1.5
	Meropenem	≤0.06–64	≤0.06	0.12	99.5	0	0.5
Citrobacter spp.^*f*^ (247)	Cefiderocol	0.004–4	0.12	0.5			
	Cefepime	≤0.06 to >64	≤0.06	1	94.3	2.0	3.6
	Ceftazidime-avibactam	≤0.06 to >64	0.12	0.5	98.4	0	1.6
	Ceftolozane-tazobactam	0.12 to >64	0.25	8	82.6	3.2	14.2
	Ciprofloxacin	≤0.12 to >8	≤0.12	1	91.5	0.4	8.1
	Colistin	≤0.25 to >8	0.5	1	99.6	0	0.4
	Meropenem	≤0.06–8	≤0.06	≤0.06	96.8	0.4	2.8
Citrobacter freundii (160)	Cefiderocol	0.004–2	0.12	0.5			
	Cefepime	≤0.06 to >64	≤0.06	2	92.5	2.5	5.0
	Ceftazidime-avibactam	≤0.06 to >64	0.12	0.5	98.1	0	1.9
	Ceftolozane-tazobactam	0.12 to >64	0.25	16	75.6	3.8	20.6
	Ciprofloxacin	≤0.12 to >8	≤0.12	4	88.1	0	11.9
	Colistin	≤0.25–2	0.5	1	100	0	0
	Meropenem	≤0.06–8	≤0.06	0.12	95.6	0.6	3.8
Citrobacter koseri (73)	Cefiderocol	0.008–4	0.25	0.5			
	Cefepime	≤0.06–8	≤0.06	≤0.06	98.6	1.4	0
	Ceftazidime-avibactam	≤0.06–1	0.12	0.25	100	0	0
	Ceftolozane-tazobactam	0.12–4	0.25	0.5	98.6	1.4	0
	Ciprofloxacin	≤0.12 to >8	≤0.12	≤0.12	98.6	0	1.4
	Colistin	≤0.25 to >8	0.5	1	98.6	0	1.4
	Meropenem	≤0.06–0.12	≤0.06	≤0.06	100	0	0
Pseudomonas aeruginosa (765)	Cefiderocol	≤0.002–4	0.12	0.5			
	Cefepime	≤0.06 to >64	4	32	82.1	7.5	10.5
	Ceftazidime-avibactam	≤0.06 to >64	2	8	91.6	0	8.4
	Ceftolozane-tazobactam	≤0.06 to >64	0.5	4	90.9	2.1	7.1
	Ciprofloxacin	≤0.12 to >8	0.25	>8	74.0	4.1	22.0
	Colistin	≤0.25 to >8	1	2	98.7	0.5	0.8
	Meropenem	≤0.06 to >64	0.5	16	73.6	5.2	21.2
Meropenem-nonsusceptible Pseudomonas aeruginosa (202)	Cefiderocol	0.008–4	0.25	1			
	Cefepime	1 to >64	16	>64	47.5	18.3	34.2
	Ceftazidime-avibactam	1 to >64	8	64	68.3	0	31.7
	Ceftolozane-tazobactam	0.5 to >64	1	>64	67.3	5.9	26.7
	Ciprofloxacin	≤0.12 to >8	8	>8	34.7	5.0	60.4
	Colistin	≤0.25–4	1	1	99.0	1.0	0
	Meropenem	4 to >64	8	16	0	19.8	80.2
Acinetobacter baumannii (839)	Cefiderocol	0.004–64	0.12	1			
	Cefepime	≤0.06 to >64	64	>64	26.5	12.5	61.0
	Ceftazidime-avibactam	≤0.06 to >64	16	>64			
	Ceftolozane-tazobactam	≤0.06 to >64	8	64			
	Ciprofloxacin	≤0.12 to >8	>8	>8	21.9	0.1	78.0
	Colistin	≤0.25 to >8	1	>8	87.5	0	12.5
	Meropenem	≤0.06 to >64	32	>64	29.1	0.6	70.3
Meropenem-nonsusceptible Acinetobacter baumannii (595)	Cefiderocol	0.004–64	0.12	1			
	Cefepime	4 to >64	64	>64	2.4	13.6	84.0
	Ceftazidime-avibactam	1 to >64	32	>64			
	Ceftolozane-tazobactam	1 to >64	16	>64			
	Ciprofloxacin	≤0.12 to >8	>8	>8	0.2	0	99.8
	Colistin	≤0.25 to >8	1	>8	82.7	0	17.3
	Meropenem	4 to >64	64	>64	0	0.8	99.2
Stenotrophomonas maltophilia (276)	Cefiderocol	0.004–2	0.06	0.25			
	Cefepime	0.5 to >64	32	>64			
	Ceftazidime-avibactam	0.5 to >64	16	64			
	Ceftolozane-tazobactam	0.12 to >64	8	>64			
	Ciprofloxacin	≤0.12 to >8	2	8			
	Colistin	≤0.25 to >8	1	>8			
	Meropenem	0.12 to >64	>64	>64			
Burkholderia cepacia (6)	Cefiderocol	0.004–1					
	Cefepime	16 to >64					
	Ceftazidime-avibactam	2–32					
	Ceftolozane-tazobactam	1–64					
	Ciprofloxacin	0.5 to >8					
	Colistin	≤0.25 to >8					
	Meropenem	2–16			66.7	0	33.3

a MIC_50_ and MIC_90_ calculated only for genus or species where >30 isolates were tested. Species of Enterobacteriaceae with <30 isolates were grouped with the overall genus data.

b Blank spaces mean that there are no CLSI, EUCAST, or FDA MIC breakpoints available for this agent.

c The 1,021 isolates of Klebsiella spp. were composed of 761 Klebsiella pneumoniae and 260 Klebsiella oxytoca isolates.

d The 493 isolates of Serratia spp. were composed of 455 Serratia marcescens, 33 Serratia liquefaciens, 3 Serratia ureilytica, 1 Serratia odorifera, and 1 Serratia rubidaea isolates.

e The 530 isolates of Enterobacter spp. were composed of 301 Enterobacter cloacae, 204 Enterobacter aerogenes, 19 Enterobacter asburiae, 4 Enterobacter kobei, and 2 Enterobacter ludwigii isolates.

f The 247 isolates of Citrobacter spp. were composed of 160 Citrobacter freundii, 73 Citrobacter koseri, 9 Citrobacter braakii, 3 Citrobacter amalonaticus, and 2 Citrobacter farmeri isolates.

**FIG 4:**
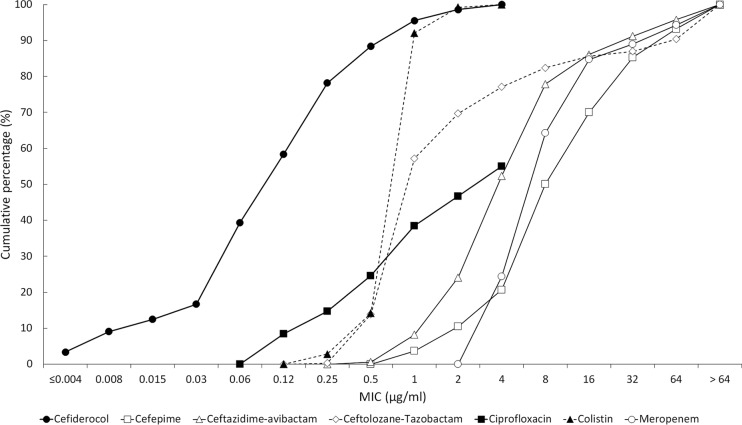
Cumulative MIC susceptibility curves for cefiderocol and comparators against 353 meropenem-nonsusceptible P. aeruginosa isolates from North American and European medical center laboratories. For colistin and ciprofloxacin, the endpoint of their respective cumulative MIC susceptibility curves represents the highest concentration tested. The remaining tested isolates had MICs higher than their endpoint value.

The MIC_90_ values for cefiderocol against P. aeruginosa were 0.5 μg/ml (North America, *n* = 765 isolates) and 0.5 μg/ml (Europe, *n* = 765 isolates); 99.9% (1,529/1,530) of all P. aeruginosa isolates had MICs of ≤4 μg/ml. The one isolate with a MIC to cefiderocol of 8 μg/ml was from North America. Against meropenem-nonsusceptible (MIC ≥ 4 μg/ml) isolates from North America (Fig. 1) and Europe (Fig. 2), MIC_90_ values for cefiderocol were 0.5 μg/ml (*n* = 151) and 1 μg/ml (*n* = 202); all 353 isolates of P. aeruginosa that were meropenem nonsusceptible had MICs to cefiderocol of ≤4 μg/ml. In comparison, the MIC_90_ values for ceftazidime-avibactam and ceftolozane-tazobactam against isolates of meropenem-nonsusceptible P. aeruginosa from North America (Table 1) and Europe (Table 2) were 8 and 4 μg/ml and 64 and >64 μg/ml, respectively. Against meropenem-susceptible (MIC ≤ 2 μg/ml) isolates from North America and Europe, MIC_90_ values for cefiderocol were 0.5 μg/ml (*n* = 614) and 0.5 μg/ml (*n* = 563); 99.9% (1,176/1,177) of all meropenem-susceptible isolates of P. aeruginosa had MICs to cefiderocol of ≤4 μg/ml. Figure 4 depicts the cumulative percentages of all meropenem-nonsusceptible isolates of P. aeruginosa that were susceptible to increasing concentrations of cefiderocol and its comparators.

**FIG 5:**
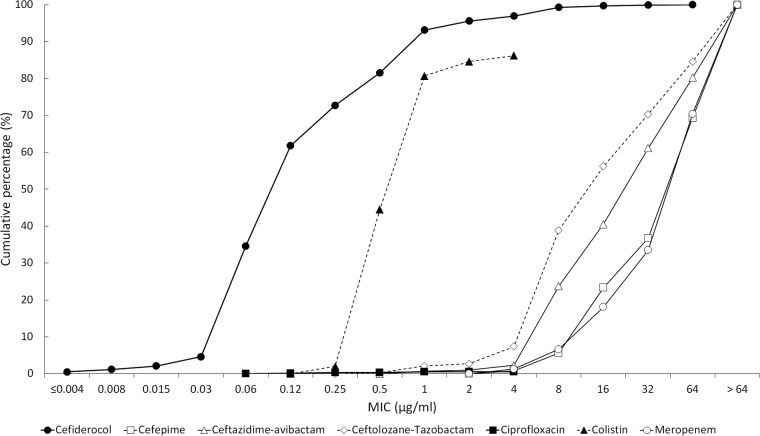
Cumulative MIC susceptibility curves for cefiderocol and comparators against 768 meropenem-nonsusceptible A. baumannii isolates from European and North American medical center laboratories. For colistin and ciprofloxacin, the endpoint of their respective cumulative MIC susceptibility curves represents the highest concentration tested. The remaining tested isolates had MICs higher than their endpoint value.

The MIC_90_ values for cefiderocol against A. baumannii were 1 μg/ml for both North American (*n* = 309) and European (*n* = 839) isolates; 97.6% (1,120/1,148) of all A. baumannii had MICs to cefiderocol of ≤4 μg/ml. Of the 28 isolates with cefiderocol MIC values of >4 μg/ml, 25 were from European medical laboratories (18 isolates from two sites in Russia; 6 isolates from one site in Turkey; and 1 isolate from Sweden) and 3 isolates were from two sites in the United States. Twenty-four of these 28 isolates (85.7%) were also nonsusceptible to meropenem. Against meropenem-nonsusceptible isolates of A. baumannii from North America (Fig. 1; *n* = 173) and Europe (Fig. 2; *n* = 595), MIC_90_ values were 1 μg/ml for both data sets; 96.9% (744/768) of meropenem-nonsusceptible A. baumannii isolates had MICs to cefiderocol of ≤4 μg/ml. Against meropenem-susceptible isolates from North America and Europe, MIC_90_ values for cefiderocol were 0.25 μg/ml (*n* = 136) and 0.25 μg/ml (*n* = 244); 99.0% (376/380) of meropenem-nonsusceptible A. baumannii isolates had MICs to cefiderocol of ≤4 μg/ml. Of the other agents tested against isolates of A. baumannii, colistin was the only one demonstrating significant *in vitro* activity. Figure 5 depicts the cumulative percentages of all meropenem-nonsusceptible isolates of A. baumannii that were susceptible to increasing concentrations of cefiderocol and its comparators.

The MIC_90_ values for cefiderocol against Stenotrophomonas maltophilia were 0.5 μg/ml (North America, *n* = 152 isolates) and 0.25 μg/ml (Europe, *n* = 276 isolates); all isolates of S. maltophilia had MICs to cefiderocol of ≤4 μg/ml, while the MIC_90_s for cefepime, ceftazidime-avibactam, ceftolozane-tazobactam, and meropenem were ≥64 μg/ml and for colistin and ciprofloxacin were ≥8 μg/ml. There are no published CLSI breakpoints for S. maltophilia for any of the other antimicrobial agents tested in this study.

The MIC range for cefiderocol for 12 isolates of Burkholderia cepacia was 0.008 to 16 μg/ml. Three isolates were meropenem nonsusceptible (MIC ≥ 8 μg/ml). Too few isolates were collected to generate MIC_50_ and MIC_90_ values. One isolate from the United States had a cefiderocol MIC of 16 μg/ml; all other isolates (93.8%, 11/12) had cefiderocol MICs of ≤1 μg/ml.

If the entire data set is considered (*n* = 9,205 isolates) and species intrinsically resistant to colistin (B. cepacia, Serratia spp.) and species for which colistin MIC breakpoints are not available (S. maltophilia) are excluded, colistin nonsusceptibility was observed for 273 isolates (121 A. baumannii, 73 Enterobacter species, 57 Klebsiella species, 14 P. aeruginosa, 6 E. coli, and 2 Citrobacter koseri isolates). The cefiderocol MIC range and MIC_90_ for colistin-nonsusceptible isolates were ≤0.002 to 8 μg/ml and 2 μg/ml, respectively; 99.6% (272/273) isolates had cefiderocol MICs of ≤4 μg/ml.

## DISCUSSION

Carbapenem-resistant Enterobacteriaceae, P. aeruginosa, and A. baumannii are frequently multidrug resistant. Currently, there are very few antimicrobial agents available to clinicians to treat patients infected with carbapenem-resistant Gram-negative bacilli, and the few agents that are accessible to treat systemic infections are associated with considerable toxicities, increasing resistance, and in the case of colistin, intrinsic resistance to several species of Enterobacteriaceae, including Proteus spp., Providencia spp., Morganella morganii, and Serratia spp. In addition, new β-lactam/β-lactamase inhibitor combinations, ceftazidime-avibactam and ceftolozane-tazobactam, have recently been approved in several countries, but neither of these agents is active against isolates producing class B metallo-β-lactamases (1, 2). In the current study, meropenem-nonsusceptible isolates of Enterobacteriaceae and P. aeruginosa challenged the *in vitro* activities of ceftazidime-avibactam and ceftolozane-tazobactam, particularly isolates from European medical center laboratories (Table 2).

The current study demonstrated that the *in vitro* activity of the novel siderophore cephalosporin, cefiderocol, was superior to that of comparators against recent clinical isolates of meropenem-nonsusceptible Enterobacteriaceae, P. aeruginosa, and A. baumannii from North America and Europe (Fig. 3 to 5), including isolates that were resistant to colistin and the β-lactam/β-lactamase inhibitor combinations ceftazidime-avibactam and ceftolozane-tazobactam. Cefiderocol also demonstrated potent activity against S. maltophilia, while all six comparators were inactive. Cefiderocol exhibited MIC_90_s against P. aeruginosa and S. maltophilia that were 4 to >64 times lower than those of comparator agents. Against A. baumannii, cefiderocol (MIC_90_, 1 μg/ml) was up to 64 times more potent than the comparator agents tested, with the exception of colistin, which also had an MIC_90_ of 1 μg/ml.

Cefiderocol at a concentration of ≤4 μg/ml inhibited 99.9% (6,078/6,087) of all isolates of Enterobacteriaceae, 99.9% (1,529/1,530) of all isolates of P. aeruginosa, 97.6% (1,120/1,148) of all isolates of A. baumannii, and 100% (428/428) of all isolates of S. maltophilia. The highest MIC for cefiderocol for Enterobacteriaceae (seven isolates) and P. aeruginosa (one isolate) was 8 μg/ml. Against A. baumannii, 28 isolates (2.3%) had MICs of >4 μg/ml. One isolate of Burkholderia cepacia, of the 12 tested, had a cefiderocol MIC of 16 μg/ml (from the United States); all other isolates had cefiderocol MICs of ≤1 μg/ml.

Previous studies have tested the *in vitro* activity of cefiderocol against genetically characterized ESBL- and carbapenemase-producing isolates of Gram-negative bacilli as well as against isolates of Gram-negative bacilli resistant to carbapenems by mechanisms other than carbapenemases (15–17, 24). Isolates of E. coli, K. pneumoniae, S. marcescens, Citrobacter freundii, and Enterobacter cloacae harboring ESBLs (e.g., CTX-type, SHV-type, TEM-type), KPC-type carbapenemases, and OXA-type carbapenemases as well as K. pneumoniae, S. marcescens, C. freundii, and E. cloacae harboring VIM-type and IMP-type carbapenemases were all inhibited by cefiderocol at a MIC of ≤4 μg/ml (15). Most NDM-1-producing isolates of E. coli (73.7%, 14/19), K. pneumoniae (100%, 24/24), and S. marcescens, C. freundii, and E. cloacae (100%, 6/6) also tested with MICs to cefiderocol of ≤4 μg/ml (15). Ito et al. reported that among 33 isolates of P. aeruginosa, MICs to cefiderocol were ≤2 μg/ml for isolates harboring GIM-1, IMP-type, or SPM-1 carbapenemases and that 14 of 16 (87.5%) VIM-positive isolates had a MIC to cefiderocol of ≤4 μg/ml (17). Against 29 isolates of A. baumannii shown to be β-lactamase-producing, MICs to cefiderocol were ≤4 μg/ml for isolates harboring IMP-1, OXA-51, and OXA-58; some isolates that were positive for OXA-23 or OXA-24 were less susceptible to cefiderocol than were other OXA-type-positive isolates (17).

The mechanism(s) responsible for elevated MICs for cefiderocol in the limited number of isolates that have been observed are currently unknown. If one were to hypothesize a mechanism of resistance to cefiderocol, it would likely involve either a reduction in production of one or more components of the iron transport system or one or more mutations in the binding site for the iron transport system on the outer membrane of Gram-negative bacteria, as this has been reported previously for other siderophore β-lactams (25, 26). The adaption-based resistance to other siderophore-conjugated antibacterial agents, such as MB-1, attributed to competition with native siderophores in P. aeruginosa (26), has not been observed for cefiderocol tested against isolates of P. aeruginosa, A. baumannii, or Enterobacteriaceae (21). The mechanism(s) of resistance to cefiderocol that appeared in the small subset of isolates with higher MICs to cefiderocol (≥8 μg/ml) in the current study requires future study.

Cefiderocol has been tested in several animal models and has shown *in vivo* efficacy against ESBL-producing, KPC-producing, and multidrug-resistant isolates of Gram-negative bacilli (21, 23, 27). In a neutropenic murine thigh infection model, cefiderocol treatment consistently generated CFU reductions of >1 log and sustained antibacterial effects against all isolates tested (23). In the same study, the effectiveness of cefiderocol was demonstrated to correlate well with the pharmacodynamics parameter % *f*T>MIC (the percentage of a 24-hour period in which the unbound drug concentration exceeded the MIC) (23). Cefiderocol has also demonstrated efficacy against multidrug-resistant and carbapenem-resistant isolates of P. aeruginosa, A. baumannii, and Enterobacteriaceae in a murine lung infection model (21) and against ESBL-producing or KPC-producing isolates of Enterobacteriaceae in various animal infection models (27). In pharmacokinetic modeling of human drug exposures using a dose of 2 g every 8 h infused over 3 h, cefiderocol achieves a 100% probability to target attainment for organisms with an MIC of 4 μg/ml (28). Importantly, in a rat lung infection model that reproduced human pharmacokinetic and pharmacodynamics parameters, cefiderocol demonstrated bactericidal activity against carbapenem-resistant (KPC- and NDM-1-positive) isolates of K. pneumoniae, with cefiderocol MICs as high as 4 μg/ml (29). A clinical trial evaluating the efficacy and safety of cefiderocol versus imipenem or cilastatin in complicated urinary tract infections is currently ongoing (30) and will be followed by a clinical trial comparing cefiderocol to the best available therapies for the treatment of serious infections caused by carbapenem-resistant Gram-negative pathogens (31).

The current study tested cefiderocol, a promising, novel siderophore cephalosporin, using the CLSI-approved broth microdilution method with ID-CAMHB prepared by preincubation with Chelex 100 resin (20), and found it to demonstrate potent *in vitro* activity against Enterobacteriaceae, P. aeruginosa, A. baumannii, S. maltophila, and B. cepacia, including carbapenem-resistant isolates. A cefiderocol MIC of ≤4 μg/ml was observed for 99.6% (9,166/9,205) of all isolates of Gram-negative bacilli tested in the current study. Previous studies that performed molecular characterization of isolates for β-lactamase genes have reported that cefiderocol is stable against a broad range of β-lactamases, including ESBL-, AmpC-, and carbapenemase-producing (class A, B, and D enzymes) isolates, including metallo-β-lactamases (e.g., NDM-1, VIM, IMP), as well as against isolates resistant to carbapenems by alternate mechanisms (15–17, 24). Cefiderocol represents a potentially significant advance in the treatment options available to clinicians to care for patients infected with antimicrobial-resistant Gram-negative bacilli. Further *in vitro* and clinical work with cefiderocol is warranted.

## MATERIALS AND METHODS

### Bacterial isolates.

From 1 November 2014 to 31 October 2015, 99 medical center laboratories from 13 countries (Canada, 9 medical center laboratories; United States, 41; Czech Republic, 3; France, 5; Germany, 6; Greece, 4; Hungary, 4; Italy, 5; Russia, 5; Spain, 5; Sweden, 2; Turkey, 5; United Kingdom, 5) were each requested to collect 100 clinical isolates with a specific species distribution (15 for E. coli, 15 for K. pneumoniae, 5 for Klebsiella spp. other than K. pneumoniae, 10 for Enterobacter spp., 10 for Serratia spp., 5 for Citrobacter spp., 15 for P. aeruginosa, 15 for A. baumannii, 5 for Burkholderia cepacia, and 5 for Stenotrophomonas maltophilia) from patients with documented intra-abdominal, urinary tract, skin and soft tissue, lower respiratory tract, or bloodstream infections. Only one isolate per patient infection episode was accepted. All isolates were shipped to International Health Management Associates, Inc. (IHMA, Schaumburg, IL, USA), where their identities were confirmed using matrix-assisted laser desorption ionization–time of flight mass spectrometry (MALDI-TOF MS; Bruker Daltonics, Billerica, MA, USA). In total, 9,205 isolates of Gram-negative bacilli were collected by the 50 medical center laboratories in North America (*n* = 4,239) and the 49 medical center laboratories in Europe (*n* = 4,966).

### Antimicrobial susceptibility testing.

All aspects of antimicrobial susceptibility testing, including broth microdilution panel production, inoculation, incubation, and interpretation, adhered to current CLSI methods (32, 33) and were conducted by IHMA. Broth microdilution panels included the following antimicrobial agents: cefiderocol (doubling dilution range tested, 0.002 to 256 μg/ml), cefepime (0.06 to 64 μg/ml), ceftazidime-avibactam (0.06 and 4 μg/ml to 64 and 4 μg/ml), ceftolozane-tazobactam (0.06 and 8 μg/ml to 64 and 8 μg/ml), ciprofloxacin (0.12 to 8 μg/ml), colistin (0.25 to 8 μg/ml), and meropenem (0.06 to 64 μg/ml). Cefiderocol and ceftolozane were obtained from Shionogi & Co., Ltd. (Osaka, Japan). Avibactam was obtained from Biochempartner (Wuhan, China). Other antimicrobial agents were obtained from the U.S. Pharmacopeia (Rockville, MD). All antimicrobial agents were dissolved and diluted according to CLSI guidelines (20, 32). Cefiderocol was dissolved and diluted in sterile normal saline (20). CAMHB was prepared according to the manufacturer's (BBL, Becton-Dickinson, Sparks, MD) instructions and was used as recommended by the CLSI for broth microdilution panel preparation (32, 33). Cefiderocol was tested using ID-CAMHB prepared by adding 100 g of Chelex 100 resin (Bio-Rad Laboratories, Hercules, CA) to 1 liter of autoclaved CAMHB and stirred for 2 h at room temperature (23°C) to remove cations in the medium. The iron-depleted broth was then filtered using a 0.2-μm filter, its pH was adjusted to 7.3 using 0.1 M hydrochloric acid, and then the broth was supplemented with calcium (CaCl_2_), magnesium (MgCl_2_), and zinc (ZnSO_4_) to final concentrations of 22.5 mg/liter (range, 20 to 25 mg/liter), 11.25 mg/liter (range, 10 to 12.5 mg/liter), and 10 μM (0.56 mg/liter; range 0.5 to 1.0 mg/liter), respectively, and again passed through a 0.2-μm filter. The method of preparation of ID-CAMHB described above was approved by the CLSI Subcommittee on Antimicrobial Susceptibility Testing in January 2016 (20, 34) and has supplanted previous medium preparation methods, including those using 20 μM human apotransferrin and Chelex-treated Iso-Sensitest broth due to MIC reproducibility issues or to the very limited number of manufacturers able to provide media (15–17, 24). The final concentration of iron in ID-CAMHB prepared using the above-described method is ≤0.03 mg/liter (20).

The broth microdilution panels included growth control wells for both CAMHB and ID-CAMHB. The panels were incubated at 35°C for 20 h in ambient air before MIC endpoints were read. ID-CAMHB did not significantly affect the growth of any quality control or test organism. Reading the MIC of cefiderocol was contingent on the presence of strong growth in the ID-CAMHB growth control (i.e., a button of approximately 2 mm or greater). The cefiderocol MIC was read as the first panel well in which isolate growth was significantly reduced (i.e., a button of <1 mm or light/faint turbidity) relative to the growth observed in the ID-CAMHB growth control well. The method described above for reading MIC endpoints for cefiderocol was approved by the CLSI Subcommittee on Antimicrobial Susceptibility Testing in January 2016 (20, 34).

CLSI interpretive criteria, when available (32), and FDA interpretive criteria for ceftazidime-avibactam (35) were used to interpret MICs. For colistin, CLSI interpretive criteria were used to interpret colistin MICs for P. aeruginosa and A. baumannii. Colistin lacks CLSI or FDA breakpoints for Enterobacteriaceae; therefore, European Committee on Antimicrobial Susceptibility Testing (EUCAST) MIC breakpoints for Enterobacteriaceae were applied to Enterobacteriaceae tested against colistin (susceptible, ≤2 μg/ml; resistant, ≥4 μg/ml) (36). Quality control testing was performed each day of testing using E. coli ATCC 25922, P. aeruginosa ATCC 27853, and K. pneumoniae ATCC 700603. All quality control results were within specified CLSI ranges (32), including the CLSI-approved but not-yet-published ranges for cefiderocol (E. coli ATCC 25922, 0.06 to 0.5 μg/ml; P. aeruginosa ATCC 27853, 0.06 to 0.5 μg/ml) (20).
